# One-Stage Versus Two-Stage ACL Reconstruction with Concomitant MCL Surgery in Combined ACL and MCL Injuries: A Minimum 2-Year Follow-Up Study

**DOI:** 10.3390/jcm15020583

**Published:** 2026-01-11

**Authors:** Kwangho Chung, Hyun-Soo Moon, Sung-Hwan Kim, Seung Ho Yoon, Min Jung

**Affiliations:** 1 Arthroscopy and Joint Research Institute, Yonsei University College of Medicine, Seoul 03722, Republic of Korea; khchung85@gmail.com (K.C.); osmhs@yuhs.ac (H.-S.M.); orthohwan@gmail.com (S.-H.K.); 2 Department of Orthopaedic Surgery, Yongin Severance Hospital, Yonsei University College of Medicine, Yongin 16995, Republic of Korea; 3 Department of Orthopaedic Surgery, Gangnam Severance Hospital, Yonsei University College of Medicine, Seoul 06273, Republic of Korea; 4 Department of Orthopaedic Surgery, Severance Hospital, Yonsei University College of Medicine, Seoul 03722, Republic of Korea; syoon@yuhs.ac

**Keywords:** anterior cruciate ligament reconstruction, medial collateral ligament, combined injury, one stage, two stage

## Abstract

**Background:** The optimal timing and staging of anterior cruciate ligament reconstruction (ACLR) in patients with concomitant medial collateral ligament (MCL) injury remain controversial. This study aimed to compare clinical outcomes between a one-stage ACLR group and a two-stage ACLR group in patients with combined ACL and MCL injuries in which the MCL was surgically managed. **Methods:** This retrospective study included 68 patients with combined ACL and grade III MCL injuries treated with ACLR and MCL surgery. Patients were divided into the one-stage ACLR group (*n* = 42) and the two-stage ACLR group (*n* = 26) according to the timing and staging of ACLR relative to MCL surgery. Clinical outcomes, including knee stability, patient-reported outcomes, and range of motion (ROM), were compared between groups. **Results:** After a minimum 2-year follow-up, both groups demonstrated significant improvements in clinical and stability outcomes, with enhanced anterior knee stability, improved patient-reported outcomes, and better objectively assessed knee function. No significant differences were found between groups in anterior, valgus (one-stage: 1.8 ± 1.1 mm, two-stage: 2.3 ± 1.3 mm; *p* = 0.160), or rotational stability. Likewise, there were no significant differences in mean flexion deficits (one-stage: 2.6 ± 4.1°, two-stage: 1.0 ± 2.0°; *p* = 0.137), mean extension deficits (one-stage: 1.5 ± 2.5°, two-stage: 1.3 ± 2.0°; *p* = 0.137), flexion deficits ≥10° (one-stage: 9.5% [4/42], two-stage: 0%; *p* = 0.290), extension deficits ≥ 5° (one-stage: 9.5% [4/42], two-stage: 3.8% [1/26]; *p* = 0.642), or additional procedures for postoperative stiffness (one-stage: 16.7% [7/42], two-stage: 11.5% [3/26]; *p* = 0.730). Patient-reported outcomes, including the Lysholm and IKDC subjective scores, were also comparable between groups. **Conclusions:** Both the one-stage ACLR group and the two-stage ACLR group for surgically managed combined ACL and MCL injuries yielded comparable clinical and stability outcomes, suggesting that one-stage ACLR can be performed without an apparent increase in the risk of postoperative stiffness or ROM limitations. However, given the limited sample size, these results should be interpreted cautiously because the study may have been insufficiently powered to detect small clinically meaningful differences.

## 1. Introduction

Combined anterior cruciate ligament (ACL) and medial collateral ligament (MCL) injuries are relatively common, occurring in up to 38% of all ACL injuries [1,2]. These combined injuries are biomechanically important because the ACL and MCL function synergistically to maintain knee stability. Deficiency of the MCL increases the load on the ACL under both valgus stress and anterior tibial translation [3]. Consequently, if not appropriately managed, combined ACL and MCL injuries may result in inferior clinical outcomes after ACL reconstruction (ACLR), including lower patient-reported outcome scores and higher failure rates compared with isolated ACL injuries [2,4].

ACL injuries causing instability in patients with combined ACL and MCL injuries generally require ACLR regardless of the grade of MCL injury, particularly in young and active individuals [5]. Most concomitant MCL injuries, especially partial ruptures classified as grade I or II, are typically managed nonoperatively, whereas surgical intervention is usually reserved for complete grade III MCL tears [6,7,8]. However, no clear consensus exists regarding the optimal treatment strategy for such combined injuries [2,9,10].

The optimal timing and staging of ACLR in cases of acute combined ACL and MCL injuries remain major points of controversy. A one-stage approach, involving simultaneous reconstruction of both ligaments, may theoretically restore knee stability and facilitate quicker rehabilitation; however, it has also been associated with an increased risk of arthrofibrosis and limited range of motion [8,11]. In contrast, a two-stage approach, in which ACLR is delayed following initial MCL surgery or healing, allows for recovery of knee motion before ACLR, but may lead to persistent valgus laxity, delayed return to sports, and a greater risk of intra-articular damage [12,13]. Despite these theoretical advantages and disadvantages, few studies have directly compared one-stage and two-stage ACLR in the setting of surgically managed MCL injuries.

The purpose of this study was to compare postoperative clinical outcomes between a one-stage ACLR group and a two-stage ACLR group in patients with combined ACL and MCL injuries in which the MCL was surgically managed. We hypothesized that one-stage ACLR would be associated with better knee stability and patient-reported outcomes but a higher risk of postoperative stiffness and ROM deficits compared with two-stage ACLR.

## 2. Materials and Methods

### 2.1. Study Population

**Figure 1:**
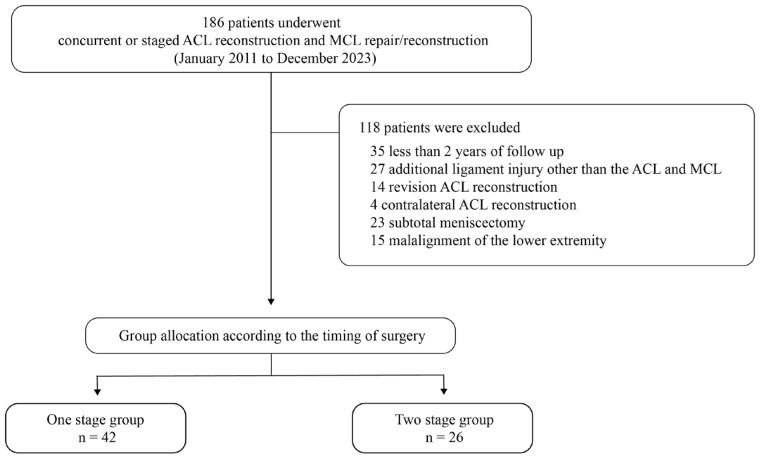
Study flow diagram depicting patient selection.

We received the revision decision letter for our initial submission, which was conducted under the original Institutional Review Board (IRB) approval, on 20 November 2025. The letter recommended expanding the patient inclusion period. Accordingly, we applied for an updated IRB approval and submitted additional documents on 24 November 2025; approval was granted on 10 December 2025. After obtaining IRB approval, we ultimately performed a retrospective review of patients who underwent combined ACL and MCL surgery between January 2011 and December 2023. Inclusion criteria were as follows: (1) single-bundle ACLR; (2) concomitant MCL surgery (repair or reconstruction) for a grade III injury, defined as a complete rupture identified on preoperative MRI [14]; and (3) a minimum follow-up duration of 2 years. The exclusion criteria were as follows: (1) additional ligament injuries requiring surgery other than the ACL and MCL; (2) revision ACLR; (3) contralateral ACLR; (4) subtotal or total meniscectomy for meniscal tears; (5) skeletal immaturity; (6) prior surgical procedures involving the ipsilateral knee; and (7) coronal plane malalignment of the lower extremity, defined as a mechanical axis line passing more than 8 ± 7 mm medial to the center of the knee joint line on standing hip–knee–ankle radiographs [15]. Overall, 68 patients satisfied the inclusion criteria and were included in the final analysis (Figure 1). Based on the timing and staging of MCL surgery relative to ACLR, patients were divided into the one-stage ACLR group (*n* = 42) and the two-stage ACLR group (*n* = 26).

### 2.2. Operative Procedures

Whether to proceed with one-stage or two-stage ACLR was determined after a detailed discussion with each patient regarding the potential advantages and disadvantages of both surgical approaches. In the one-stage ACLR group, ACLR and MCL repair or reconstruction were performed concurrently, whereas in the two-stage ACLR group, ACLR was performed approximately 8 weeks after the initial MCL surgery, once full range of motion had been restored.

Diagnostic arthroscopy was performed first to confirm ACL rupture before proceeding with ACLR and to identify any concomitant intra-articular pathology. Concomitant meniscal tears were treated appropriately based on tear characteristics before ligament reconstruction. Subsequently, ACLR was performed. Following graft preparation, the tibial and femoral tunnels were drilled at the respective anatomic ACL footprints based on the graft diameter. The graft was subsequently introduced and fixed within both tunnels.

Surgical strategy for medial collateral ligament injuries was determined based on tear location, tissue quality, and intraoperative assessment of valgus stability. Primary repair was performed when the lesion demonstrated a repairable pattern, such as femoral or tibial avulsion with adequate tissue quality or soft-tissue interposition preventing anatomic healing. Reconstruction was selected in cases of midsubstance disruption with poor tissue integrity or when adequate valgus stability could not be restored with direct repair alone [16]. For MCL repair, after identifying the tear site, the torn ligament was repaired using suture anchors or staples at the proximal or distal MCL insertion, depending on the location of the tear. If primary repair was not feasible because of the tear pattern, MCL reconstruction was performed instead. For MCL reconstruction, the semitendinosus tendon, with its tibial attachment preserved, was used as the graft. The femoral isometric point was identified near the medial femoral epicondyle, and the graft was looped around the shank of a screw and washer for fixation at 30° of knee flexion with a varus force applied to reproduce the native function of the MCL [17]. The free end of the graft was then routed posteriorly beneath the insertion of the semimembranosus tendon and fixed at the tibial insertion of the direct head of the semimembranosus tendon to reinforce the posterior oblique ligament.

### 2.3. Postoperative Rehabilitation

The same postoperative rehabilitation protocol was applied to all patients. From immediately after surgery until 8 weeks postoperatively, patients ambulated with crutches and used a knee brace. The knee was immobilized for the first 2 weeks after surgery. Beginning at 2 weeks postoperatively, gradual range-of-motion exercises were initiated, with a target of achieving 90° of flexion by 4 weeks and full range of motion by 8 weeks. At the 2-week postoperative outpatient visit, patients were instructed on proper brace use and home-based range-of-motion exercises, and, depending on the degree of ROM recovery, supervised physiotherapy at the outpatient clinic was provided up to three times per week. Partial weight-bearing with crutch assistance was allowed immediately after surgery, and isometric quadriceps-strengthening exercises were encouraged. In the two-stage ACLR group, patients followed the same rehabilitation protocol after the first surgery, and the identical protocol was reapplied following the second surgery. Swimming, cycling, and jogging were allowed 12 weeks after the final surgery, while return to sports requiring sidestepping, jumping, or pivoting was permitted at 6 months.

### 2.4. Clinical and Radiologic Evaluation

The primary outcome of this study was postoperative anterior and medial knee stability. Secondary outcomes included postoperative range of motion, stiffness-related secondary procedures, patient-reported outcomes, and radiographic degenerative changes. Clinical outcomes were evaluated preoperatively and at final follow-ups after surgery. Anterior knee stability was assessed using the Lachman test and an instrumented KT-2000 arthrometer (Medmetric, San Diego, CA, USA) with the knee flexed to 30° under a 134-N anterior load. The side-to-side difference between the affected and contralateral knees was recorded, and the Lachman test results were graded as grade 0 (<3 mm), grade 1 (3–5 mm), grade 2 (6–10 mm), or grade 3 (>10 mm). Medial stability was evaluated using valgus stress radiographs obtained with the knee flexed to 20° using a Telos stress device (Telometer; Daiseung Medics, Seoul, Republic of Korea). With the patient in a relaxed supine position, a valgus stress was applied at the joint line. Two medial support pads were placed 20 cm proximal and 20 cm distal to the joint line. Medial joint opening was defined as the minimal distance between the subchondral bone of the medial femoral condyle and that of the medial tibial plateau [18]. Side-to-side differences were calculated as the contralateral value minus the affected-side value. Preoperative valgus stress radiographs were not obtained to avoid the risk of iatrogenic MCL injury. Two blinded orthopedic fellows independently performed the radiographic measurements, and the average of their measurements was used for analysis.

Functional outcomes were assessed preoperatively and postoperatively using the Lysholm knee score [19], the subjective International Knee Documentation Committee (IKDC) score, and the objective IKDC examination form [20]. Degenerative changes were assessed on standardized radiographs, including anteroposterior, lateral, posteroanterior 45° weight-bearing, and Merchant views. Arthritic grading was determined according to the IKDC radiographic assessment scale [20].

### 2.5. Statistical Analysis

We performed the statistical analyses using SPSS version 26.0 (IBM Corp., Armonk, NY, USA) and assessed distributional normality with the Shapiro–Wilk test. Normally distributed continuous variables were compared between groups using an independent Student’s *t*-test, whereas non-normally distributed variables were evaluated using the Mann–Whitney *U* test. Categorical variables were compared using the chi-square test or Fisher’s exact test, as appropriate.

Comparisons of pre- and postoperative changes in clinical and stability outcomes within each group were performed using the paired *t*-test for normally distributed continuous variables and the Wilcoxon signed-rank test for nonparametric continuous or ordinal variables. Because the two groups differed in MCL procedure type and duration from injury to ACLR, additional adjusted analyses were performed. Continuous postoperative outcomes were evaluated using analysis of covariance (ANCOVA), and categorical outcomes were analyzed using logistic regression to assess whether group differences persisted after adjustment for these potential confounders. Continuous data are presented as mean ± standard deviation, whereas categorical data are summarized as counts and percentages. Statistical significance was defined as *p*-value < 0.05.

## 3. Results

Baseline characteristics were comparable between the one-stage ACLR group and the two-stage ACLR group in terms of age, sex, body mass index, injury mechanism, and time from injury to the index surgery (Table 1). However, the time from injury to ACLR differed significantly between the groups (one-stage ACLR group, 5.9 ± 4.6 weeks; two-stage ACLR group, 12.1 ± 2.2 weeks; *p* < 0.001).

Preoperative clinical and radiographic evaluations revealed no significant differences between the groups in anterior knee laxity, patient-reported outcome scores, including the Lysholm and IKDC subjective scores, or IKDC radiographic grades (Table 2). Regarding MCL procedures, reconstruction and repair were performed in similar proportions in the one-stage ACLR group, whereas repair was considerably more common in the two-stage ACLR group (*p* < 0.001).

Surgical characteristics of the two groups are summarized in Table 3. Regarding ACLR, graft type, graft diameter, and femoral fixation method did not differ significantly between the two groups (*p* > 0.05). Similarly, the prevalence of concomitant meniscal tears and the choice of meniscal treatment showed no significant differences. In contrast, the distribution of MCL procedures differed markedly (*p* < 0.001): the one-stage ACLR group more frequently underwent MCL reconstruction, whereas MCL repair was considerably more common in the two-stage ACLR group.

After a minimum 2-year follow-up (one-stage ACLR group, 2.7 ± 0.3 years; two-stage ACLR group, 2.8 ± 0.5 years; *p* = 0.322), no statistically significant differences were detected between groups in anterior, valgus, or rotational stability, as indicated by the side-to-side difference (SSD) in anterior translation, the SSD in medial joint opening, and the distribution of pivot-shift grades, respectively (Table 4). In addition, there were no significant differences between the two groups in mean flexion or extension deficits, in the proportions of patients with flexion deficits of ≥10° or extension deficits of ≥5°, or in the rates of additional procedures performed for postoperative stiffness. Patient-reported outcomes, including the Lysholm and IKDC subjective scores, also showed no statistically significant difference between groups. According to additional adjusted analyses using ANCOVA and logistic regression (Table 5 and Table 6) that were performed to address potential confounding by MCL procedure type and time from injury to ACLR, neither surgical staging (one- vs. two-stage), MCL repair versus reconstruction, nor duration from injury to ACLR showed a significant association with SSD of anterior translation, SSD of medial joint opening, or flexion/extension deficits (all *p* > 0.05). Likewise, the adjusted logistic regression models did not demonstrate significant associations between these covariates and categorical outcomes, including Lachman and pivot-shift grades, flexion deficit ≥ 10°, extension deficit ≥ 5°, or stiffness-related secondary procedures. When pre- and postoperative changes in clinical and stability outcomes were compared within each group, both groups showed significant improvements in stability and clinical outcomes, including patient-reported outcomes and objectively assessed knee function, while radiographic deterioration was not observed (Table 7).

## 4. Discussion

The key finding of the study was that both one-stage and two-stage ACLR for surgically managed combined ACL and MCL injuries yielded comparable clinical and stability outcomes at a minimum follow-up of 2 years. Although flexion deficits greater than 10° and stiffness-related secondary procedures were more frequent in the one-stage ACLR group, these differences did not reach statistical significance. These results indicate that both approaches achieve similar short-term outcomes and that one-stage ACLR can be performed with minimal concern for postoperative motion limitation.

The optimal timing of ACLR in combined ACL and MCL injuries remains a matter of debate. Despite the clinical importance of this issue, only one study to date has directly compared early and delayed ACLR in the presence of concomitant MCL injury [21]. Petersen and Laprell reported similar knee stability and ROM between early (within 3 weeks after injury) and delayed (10–12 weeks after injury) ACLR when the MCL was treated nonoperatively, but higher Lysholm scores and fewer stiffness-related reoperations in the delayed group (early group, 4/27; delayed group, 1/37) [21]. However, another recent retrospective cohort study examining the timing of ACLR found that ACLR performed within 6 weeks of injury was independently associated with superior patient-reported outcome measures, including higher IKDC and KOOS scores, among patients whose concomitant grade III MCL injuries were managed nonoperatively [22]. Furthermore, according to a recent systematic review, early ACL stabilization in the acute setting appears to be associated with the lowest incidence of residual valgus laxity, regardless of the MCL management method [23]. These inconsistent findings among previous studies are likely attributable to variations in study design, patient selection, and MCL treatment methods. Despite this heterogeneity, our study provides unique insights, as it directly compared one-stage and two-stage ACLR in patients with surgically managed MCL injuries. Although our cohort involved surgically managed MCL injuries with different intervals from injury to ACLR (one-stage ACLR group, 5.9 ± 4.6 weeks; two-stage ACLR group, 12.1 ± 2.2 weeks; *p* < 0.001), no significant differences in stability or ROM deficits were found between the one-stage and two-stage ACLR groups. Patient-reported outcomes were also comparable between the two approaches. Taken together, the findings of the present study suggest that a one-stage ACLR with concomitant MCL surgery performed within approximately 6 weeks of injury may be a reasonable option in appropriately selected patients.

The ideal management of grade III MCL injuries associated with ACL tears remains unclear. In the past, concerns regarding range-of-motion deficits after MCL surgery and expectations of the MCL’s intrinsic healing potential led to the widespread adoption of nonoperative management, which generally yielded favorable outcomes following delayed ACLR [8]. However, a recent registry study highlighted a 70% increase in MCL surgeries from 2015 to 2020, indicating a growing trend toward surgical intervention, although approximately 90% of concomitant MCL injuries are still managed nonoperatively [2]. This increased interest in surgical treatment may be attributed to the growing recognition that concomitant MCL injury can negatively influence ACLR outcomes and increase the risk of graft failure compared with isolated ACL injury [7]. In a Swedish registry study, Svantesson et al. reported that patients with concomitant MCL injury—particularly those managed nonoperatively—had a higher risk of ACLR revision compared with those with isolated ACL injury [4]. In the same analysis, surgically managed MCL injuries did not increase the risk of ACLR revision relative to isolated ACL injuries. Considering these previous findings, it can be inferred that surgical management should be considered for grade III MCL injuries that meet specific indications, such as bony avulsion of the MCL, distal tears with poor healing potential, or persistent valgus laxity after failed nonoperative treatment, where surgical repair or reconstruction may provide superior stability and reduce the risk of secondary ACL graft failure. In our study, surgical management of the MCL in the setting of combined ACL and MCL injuries also demonstrated satisfactory clinical outcomes.

Stiffness has historically been a major concern after concurrent MCL surgery performed during ACLR, accounting for up to 44% of all postoperative stiffness cases, likely due to overly protective rehabilitation protocols involving prolonged bracing and restricted ROM [11,21]. Since the widespread adoption of delayed ACLR for combined ACL and MCL injuries, only a few studies have examined simultaneous surgical intervention for both ligaments in the acute setting since the 2000s [24,25,26,27,28,29]. A recent review article reported that acute simultaneous ACL and MCL surgeries generally result in low rates of ROM limitation and favorable clinical outcomes in terms of knee stability, ACLR failure, and patient-reported outcomes [12]. Across these studies, approximately 7% of patients exhibited flexion ROM deficits of ≥10°, and 3% required additional procedures for arthrofibrosis [24,25,26,27,28,29]. These encouraging results may reflect advances in surgical techniques and the implementation of early, motion-facilitating rehabilitation protocols. In our study, the one-stage ACLR group, who underwent simultaneous ACL and MCL surgery, demonstrated minimal ROM deficits (flexion deficit, 2.6 ± 4.1°; extension deficit, 1.5 ± 2.5°), with only 9.5% of patients exhibiting flexion deficits of ≥10°. Most achieved satisfactory motion recovery, and the final ROM outcomes were comparable to those of the two-stage ACLR group. These findings suggest that simultaneous one-stage ACL and MCL surgeries can be safely performed with minimal concern for postoperative ROM limitations. However, for clinical application, it is important to note that the absolute rate of stiffness-related procedures was numerically higher in the one-stage group than in the two-stage group (16.7% [7/42] vs. 11.5% [3/26]; absolute risk difference, 5.2%; *p* = 0.730). Although not statistically significant, this observed rate in the one-stage group appears higher than typically reported after contemporary ACLR, suggesting potential clinical relevance. Therefore, when ACL and grade III MCL injuries are treated with a one-stage procedure, surgeons should counsel patients regarding the risk of postoperative stiffness and ensure vigilant early rehabilitation and close monitoring of range-of-motion recovery.

This study has several limitations. First, its retrospective, non-randomized design and relatively small sample size may limit the generalizability of the findings and introduce potential selection bias. Second, the decision to proceed with one-stage versus two-stage surgery was made after discussion between the surgeon and the patient rather than on standardized criteria. This approach allows for the possibility that patients with more complex injuries were more likely allocated to one group and that those with poor preoperative range of motion were preferentially directed toward two-stage surgery. Therefore, selection bias cannot be fully excluded. Moreover, because the factors influencing the shared decision-making process were not systematically captured or standardized, residual unmeasured confounding cannot be excluded despite statistical adjustment. Third, although the patients were divided into one-stage and two-stage ACLR groups, the time interval from injury to surgery differed between the groups, which may have influenced the postoperative outcomes. Fourth, heterogeneity in the surgical techniques for MCL surgery, including the uneven distribution of MCL repair versus reconstruction and the discrepancy in time from injury to ACLR arising from the inherent differences between one-stage and two-stage strategies, may have acted as confounding factors influencing postoperative outcomes. To address this issue, we performed adjusted analyses using ANCOVA and logistic regression, which demonstrated that neither the type of MCL procedure nor the variation in time from injury to ACLR had a significant effect on postoperative outcomes. Nevertheless, some adjusted logistic regression models yielded wide confidence intervals and indications of sparse data bias, reflecting limited statistical power. Future prospective studies with standardized surgical protocols, uniform treatment algorithms, and larger cohorts are warranted to validate these findings. Fifth, ACLR was performed using various graft types and fixation methods, which introduces additional technical heterogeneity that may influence the outcomes. Although the distribution of these techniques did not differ significantly between the groups, their variability remains a potential confounding factor. Sixth, rehabilitation compliance was not objectively tracked in this retrospective study, and unmeasured variability in adherence to the prescribed protocol may have affected ROM recovery and stiffness-related outcomes. Seventh, the pivot-shift test was not included in the preoperative assessment of knee function because it could not be reliably performed in the presence of an MCL injury. Although the pivot-shift test serves as an essential indicator of rotational stability in ACL-deficient or reconstructed knees, its results may be unreliable or underestimated when a concomitant MCL injury is present [30]. Lastly, radiographic outcomes were assessed using the IKDC radiographic grading system, which may lack sensitivity for detecting subtle or early degenerative changes.

## 5. Conclusions

Both the one-stage ACLR group and the two-stage ACLR group for surgically managed combined ACL and MCL injuries yielded comparable clinical and stability outcomes. These findings suggest that one-stage ACLR with concomitant MCL surgery performed within 6 weeks of injury can be undertaken without an apparent increase in the risk of postoperative stiffness or ROM limitations. However, given the limited sample size, these results should be interpreted cautiously as the study may have been insufficiently powered to detect small clinically meaningful differences.

## Figures and Tables

**Table 1:** Baseline Demographic and Injury Characteristics of the Study Cohort.

Variable	One Stage ACLR Group (*n* = 42)	Two Stage ACLR Group (*n* = 26)	*p*-Value
Age (years) ^a^	32.8 ± 13.6	36.5 ± 9.0	0.136
Sex ^b^			0.459
Male	31 (73.8)	17 (65.4)	
Female	11 (26.2)	9 (34.6)	
Height (cm) ^a^	171.4 ± 8.6	170.3 ± 5.9	0.589
Weight (kg) ^a^	74.4 ± 12.2	75.7 ± 11.82	0.567
Body mass index (kg/cm^2^) ^a^	25.4 ± 4.3	26.0 ± 3.1	0.668
Affected side ^b^			0.537
Left	21 (50.0)	15 (57.7)	
Right	21 (50.0)	11 (42.3)	
Mechanism of injury ^b^			0.796
Road traffic accident	16 (38.1)	10 (38.5)	
Sports injury	22 (52.4)	15 (57.7)	
Miscellaneous	4 (9.5)	1 (3.8)	
Duration from injury to 1st operation (weeks) ^a^	5.9 ± 4.6	4.2 ± 2.0	0.477
Duration from injury to ACLR (weeks)	5.9 ± 4.6	12.1 ± 2.2	<0.001

^a^ Data are reported as mean ± standard deviation. ^b^ Data are reported as *n* (%).

**Table 2:** Comparison of preoperative variables between the two groups.

Variable	One Stage ACLR Group (*n* = 42)	Two Stage ACLR Group (*n* = 26)	*p*-Value
Lachman test ^b^			0.115
I	2 (4.8)	4 (15.4)	
II	35 (83.3)	16 (61.5)	
III	5 (11.9)	6 (23.1)	
SSD of Anterior translation (mm) ^a,c^	8.3 ± 2.0	7.8 ± 2.1	0.294
Lysholm knee score ^a^	59.9 ± 18.4	58.2 ± 18.6	0.709
IKDC subjective score ^a^	46.2 ± 12.8	50.4 ± 10.5	0.159
IKDC objective grade ^b^			0.914
A	0	0	
B	2 (4.8)	2 (7.7)	
C	31 (73.8)	18 (69.2)	
D	9 (21.4)	6 (23.1)	
IKDC radiographic grade ^b^			>0.999
A	38 (90.5)	23 (88.5)	
B	4 (9.5)	3 (11.5)	
C	0	0	
D	0	0	

SSD: Side-to-side difference; IKDC: International Knee Documentation Committee. ^a^ Data are reported as mean ± standard deviation. ^b^ Data are reported as *n* (%). ^c^ Measured using a KT-2000 arthrometer at 30° of knee flexion under a 134-N anterior load.

**Table 3:** Surgical Characteristics.

Variable	One Stage ACLR Group (*n* = 42)	Two Stage ACLR Group (*n* = 26)	*p*-Value
ACL Graft type ^b^			0.556
BPTB	8 (19.0)	3 (11.5)	
Hamstring	19 (45.2)	15 (57.7)	
Allograft	15 (35.7)	8 (30.8)	
ACL graft diameter ^a^	8.8 ± 0.8	8.6 ± 0.8	0.264
ACL graft femoral fixation			0.512
Interference screw	8 (19.0)	3 (11.5)	
Suspensory fixation device	34 (81.0)	23 (88.5)	
MCL procedure ^b^			<0.001
Repair	16 (38.1)	22 (84.6)	
Reconstruction	26 (61.9)	4 (15.4)	
Meniscal tear ^b^	23 (54.8)	15 (57.7)	0.813
Meniscal procedure ^b^			0.798
Meniscectomy	10 (23.8)	5 (19.2)	
Repair	13 (31.0)	10 (38.5)	
Cartilage injury ^b^	11 (26.2)	6 (23.1)	0.773

ACL: Anterior cruciate ligament; BPTB: Bone-patellar tendon bone; MCL: Medial collateral ligament. ^a^ Data are reported as mean ± standard deviation. ^b^ Data are reported as *n* (%).

**Table 4:** Between-Group Comparison of Postoperative Variables.

Variable	One Stage ACLR Group (*n* = 42)	Two Stage ACLR Group (*n* = 26)	*p*-Value
Lachman test ^b^			>0.999
0	29 (69.0)	19 (73.1)	
I	11 (26.2)	6 (23.1)	
II	2 (4.8)	1 (3.8)	
SSD of anterior translation ^a,d^	2.4 ± 1.6	2.3 ± 1.2	0.654
Pivot shift test ^b^			0.535
0	25 (59.5)	18 (69.2)	
I	15 (35.7)	6 (23.1)	
II	2 (4.8)	2 (7.7)	
SSD of medial joint opening (mm) ^a,c^	1.8 ± 1.1	2.3 ± 1.3	0.160
Flexion deficit (degree) ^a^	2.6 ± 4.1	1.0 ± 2.0	0.137
10° or more ^b^	4 (9.5)	0 (0)	0.290
Extension deficit (degree) ^a^	1.5 ± 2.5	1.3 ± 2.0	0.989
5° or more ^b^	4 (9.5)	1 (3.8)	0.642
Stiffness-related secondary procedure ^b^	7 (16.7)	3 (11.5)	0.730
Lysholm knee score ^b^	82.7 ± 11.1	83.8 ± 9.3	0.850
IKDC subjective score ^b^	77.9 ± 9.7	81.1 ± 11.0	0.200
IKDC objective grade ^b^			> 0.999
A	14 (33.3)	8 (30.8)	
B	23 (54.8)	15 (57.7)	
C	5 (11.9)	3 (11.5)	
D	0	0	
IKDC radiographic grade ^b^			0.690
A	34 (81.0)	20 (76.9)	
B	8 (19.0)	6 (23.1)	
C	0	0	
D	0	0	

SSD: Side-to-side difference; IKDC: International Knee Documentation Committee. ^a^ Data are reported as mean ± standard deviation. ^b^ Data are reported as *n* (%). ^c^ Measured on valgus stress radiographs obtained with a Telos stress device at 20° of knee flexion. ^d^ Measured using a KT-2000 arthrometer at 30° of knee flexion under a 134-N anterior load.

**Table 5:** Group comparison adjusted for MCL procedure type and time from injury to ACLR (ANCOVA Models).

Variable	Adjusted Model	*p*-Value
SSD of anterior translation	One vs. two stage ACLR	0.773
	MCL repair vs. reconstruction	0.767
	Duration from injury to ACLR	0.390
SSD of medial joint opening	One vs. two stage ACLR	0.488
	MCL repair vs. reconstruction	0.797
	Duration from injury to ACLR	0.793
Flexion deficit	One vs. two stage ACLR	0.649
	MCL repair vs. reconstruction	0.575
	Duration from injury to ACLR	0.243
Extension deficit	One vs. two stage ACLR	0.988
	MCL repair vs. reconstruction	0.297
	Duration from injury to ACLR	0.341

**Table 6:** Group comparison adjusted for MCL procedure type and time from injury to ACLR (logistic regression models).

Variable	Adjusted OR	95% Confidence Interval	*p*-Value
Lachman test	0.85	0.17–4.27	0.839
Pivot shift test	1.08	0.05–21.18	0.960
Flexion deficit ≥ 10°	-	-	>0.999 ^a^
Extension deficit ≥ 5°	3.96	0.050–315.89	0.538
Stiffness-related secondary procedure	2.33	0.48–11.32	0.294

^a^ Odds ratio and 95% CI were not reported because of sparse data and quasi-complete separation; the adjusted analysis was interpreted based on the *p*-value only.

**Table 7:** Comparison of Pre- and Postoperative Changes in Clinical and Stability Outcomes for Each Group.

Variable	One Stage ACLR Group (*n* = 42)	Two Stage ACLR Group (*n* = 26)
Lachman test	<0.001	<0.001
SSD of anterior translation	<0.001	<0.001
Lysholm knee score	<0.001	<0.001
IKDC subjective score	<0.001	<0.001
IKDC objective grade	<0.001	<0.001
IKDC radiographic grade	0.125	0.250

SSD: Side-to-side difference; IKDC: International Knee Documentation Committee. All values represent *p*-values for pre- to postoperative comparisons.

## Data Availability

Data are available upon request to the corresponding author.

## Abbreviations

The following abbreviations are used in this manuscript:

ACL	Anterior Cruciate Ligament
ACLR	AnteriAnterior Cruciate Ligament Reconstruction
MCL	Medial Collateral Ligament
ROM	Range of Motion
IKDC	International Knee Documentation Committee
KOOS	Knee Injury and Osteoarthritis Outcome Score
SSD	Side-to-Side Difference

## References

[1] LaPrade R.F., Wentorf F.A., Fritts H., Gundry C., Hightower C.D. (2007). A prospective magnetic resonance imaging study of the incidence of posterolateral and multiple ligament injuries in acute knee injuries presenting with a hemarthrosis. Arthrosc. J. Arthrosc. Relat. Surg..

[2] Niknam K., Goldberg D., Markes A.R., Feeley B.T., Zhang A.L., Ma C.B., Lansdown D.A. (2025). Concomitant medial collateral ligament injury increases the risk of revision anterior cruciate ligament reconstruction. Arthrosc. J. Arthrosc. Relat. Surg..

[3] Battaglia M.J., Lenhoff M.W., Ehteshami J.R., Lyman S., Provencher M.T., Wickiewicz T.L., Warren R.F. (2009). Medial collateral ligament injuries and subsequent load on the anterior cruciate ligament: A biomechanical evaluation in a cadaveric model. Am. J. Sports Med..

[4] Svantesson E., Hamrin Senorski E., Alentorn-Geli E., Westin O., Sundemo D., Grassi A., Custovic S., Samuelsson K. (2019). Increased risk of acl revision with non-surgical treatment of a concomitant medial collateral ligament injury: A study on 19,457 patients from the swedish national knee ligament registry. Knee Surg. Sports Traumatol. Arthrosc..

[5] Brophy R.H., Lowry K.J. (2023). American academy of orthopaedic surgeons clinical practice guideline summary: Management of anterior cruciate ligament injuries. J. Am. Acad. Orthop. Surg..

[6] Guenther D., Pfeiffer T., Petersen W., Imhoff A., Herbort M., Achtnich A., Stein T., Kittl C., Schoepp C., Akoto R. (2021). Treatment of combined injuries to the acl and the mcl complex: A consensus statement of the ligament injury committee of the german knee society (dkg). Orthop. J. Sports Med..

[7] Hays T.R., Barnum M.S., Levy B.A. (2025). Editorial commentary: Combined anterior cruciate ligament/medial collateral ligament injuries: Surgeons should have a low threshold to operate on the medial collateral ligament. Arthrosc. J. Arthrosc. Relat. Surg..

[8] Blaber O.K., DeFoor M.T., Aman Z.A., McDermott E.R., DePhillipo N.N., Dickens J.F., Dekker T.J. (2024). Lack of consensus on the management of medial collateral ligament tears in the setting of concomitant anterior cruciate ligament injury: A critical analysis. JBJS Rev..

[9] Floyd E.R., Tollefson L.V., Falaas K.L., Ebert N.J., Struyk G.D., Carlson G.B., LaPrade R.F. (2025). Evaluation of knee outcomes and anterior cruciate ligament graft failure when comparing medial collateral ligament reconstruction versus mcl repair in patients with multiple ligament knee injuries: A systematic review. Orthop. J. Sports Med..

[10] Ramakanth R., Sundararajan S.R., Sujith B.S.G., D’Souza T., Arumugam P., Rajasekaran S. (2025). Medial collateral ligament repair, isolated suture-tape-bracing and no repair for grade iii medial collateral ligament tears during anterior cruciate ligament reconstruction have similar outcome for combined anterior cruciate ligament with medial collateral ligament injury: A 3-arm randomized controlled trial. Arthrosc. J. Arthrosc. Relat. Surg..

[11] Harner C.D., Irrgang J.J., Paul J., Dearwater S., Fu F.H. (1992). Loss of motion after anterior cruciate ligament reconstruction. Am. J. Sports Med..

[12] Holuba K., Vermeijden H.D., Yang X.A., O’Brien R., van der List J.P., DiFelice G.S. (2023). Treating combined anterior cruciate ligament and medial collateral ligament injuries operatively in the acute setting is potentially advantageous. Arthrosc. J. Arthrosc. Relat. Surg..

[13] Kim S.H., Han S.J., Park Y.B., Kim D.H., Lee H.J., Pujol N. (2021). A systematic review comparing the results of early vs delayed ligament surgeries in single anterior cruciate ligament and multiligament knee injuries. Knee Surg. Relat. Res..

[14] Naraghi A.M., White L.M. (2016). Imaging of athletic injuries of knee ligaments and menisci: Sports imaging series. Radiology.

[15] Paley D., Herzenberg J.E., Tetsworth K., McKie J., Bhave A. (1994). Deformity planning for frontal and sagittal plane corrective osteotomies. Orthop. Clin. N. Am..

[16] Shultz C.L., Poehlein E., Morriss N.J., Green C.L., Hu J., Lander S., Amoo-Achampong K., Lau B.C. (2024). Nonoperative management, repair, or reconstruction of the medial collateral ligament in combined anterior cruciate and medial collateral ligament injuries-which is best? A systematic review and meta-analysis. Am. J. Sports Med..

[17] Kim S.J., Lee D.H., Kim T.E., Choi N.H. (2008). Concomitant reconstruction of the medial collateral and posterior oblique ligaments for medial instability of the knee. J. Bone Jt. Surg. Br..

[18] Laprade R.F., Bernhardson A.S., Griffith C.J., Macalena J.A., Wijdicks C.A. (2010). Correlation of valgus stress radiographs with medial knee ligament injuries: An in vitro biomechanical study. Am. J. Sports Med..

[19] Lysholm J., Gillquist J. (1982). Evaluation of knee ligament surgery results with special emphasis on use of a scoring scale. Am. J. Sports Med..

[20] Hefti F., Müller W., Jakob R.P., Stäubli H.U. (1993). Evaluation of knee ligament injuries with the ikdc form. Knee Surg. Sports Traumatol. Arthrosc..

[21] Petersen W., Laprell H. (1999). Combined injuries of the medial collateral ligament and the anterior cruciate ligament. Early acl reconstruction versus late acl reconstruction. Arch. Orthop. Trauma. Surg..

[22] Bacevich B.M., Hazzard S., Lustig M., Connelly S., Nukala V., Asnis P. (2025). Early surgical intervention results in better patient-reported outcomes than delayed treatment in patients undergoing anterior cruciate ligament reconstruction in the presence of concomitant medial collateral ligament injury. Arthrosc. Sports Med. Rehabil..

[23] van der List J.P., Muscott R.K., Parikh N., Waterman B.R., Trasolini N.A. (2024). Early anterior cruciate ligament treatment might be crucial for acute combined anterior cruciate ligament and medial collateral ligament injuries: A systematic review of the various treatment strategies. Arthrosc. J. Arthrosc. Relat. Surg..

[24] Halinen J., Lindahl J., Hirvensalo E., Santavirta S. (2006). Operative and nonoperative treatments of medial collateral ligament rupture with early anterior cruciate ligament reconstruction: A prospective randomized study. Am. J. Sports Med..

[25] Dong J., Wang X.F., Men X., Zhu J., Walker G.N., Zheng X.Z., Gao J.B., Chen B., Wang F., Zhang Y. (2015). Surgical treatment of acute grade iii medial collateral ligament injury combined with anterior cruciate ligament injury: Anatomic ligament repair versus triangular ligament reconstruction. Arthrosc. J. Arthrosc. Relat. Surg..

[26] Ateschrang A., Döbele S., Freude T., Stöckle U., Schröter S., Kraus T.M. (2016). Acute mcl and acl injuries: First results of minimal-invasive mcl ligament bracing with combined acl single-bundle reconstruction. Arch. Orthop. Trauma. Surg..

[27] Funchal L.F.Z., Astur D.C., Ortiz R., Cohen M. (2019). The presence of the arthroscopic “floating meniscus” sign as an indicator for surgical intervention in patients with combined anterior cruciate ligament and grade ii medial collateral ligament injury. Arthrosc. J. Arthrosc. Relat. Surg..

[28] Westermann R.W., Spindler K.P., Huston L.J., Wolf B.R. (2019). Outcomes of grade iii medial collateral ligament injuries treated concurrently with anterior cruciate ligament reconstruction: A multicenter study. Arthrosc. J. Arthrosc. Relat. Surg..

[29] Sim J.A., Na Y.G., Choi J.W., Lee B.H. (2022). Early medial reconstruction combined with severely injured medial collateral ligaments can decrease residual medial laxity in anterior cruciate ligament reconstruction. Arch. Orthop. Trauma. Surg..

[30] Tanaka M., Vyas D., Moloney G., Bedi A., Pearle A.D., Musahl V. (2012). What does it take to have a high-grade pivot shift?. Knee Surg. Sports Traumatol. Arthrosc..

